# Mechanistic Insights on Functionalization of Graphene with Ozone

**DOI:** 10.1021/acs.jpcc.3c03994

**Published:** 2023-11-07

**Authors:** Mohammad Tohidi Vahdat, Shaoxian Li, Shiqi Huang, Luc Bondaz, Nicéphore Bonnet, Kuang-Jung Hsu, Nicola Marzari, Kumar Varoon Agrawal

**Affiliations:** † Laboratory of Advanced Separations (LAS), École Polytechnique Fédérale de Lausanne (EPFL), Sion CH-1950, Switzerland; ‡ Theory and Simulation of Materials (THEOS) and National Centre for Computational Design and Discovery of Novel Materials (MARVEL), EPFL, Lausanne CH-1015, Switzerland

## Abstract

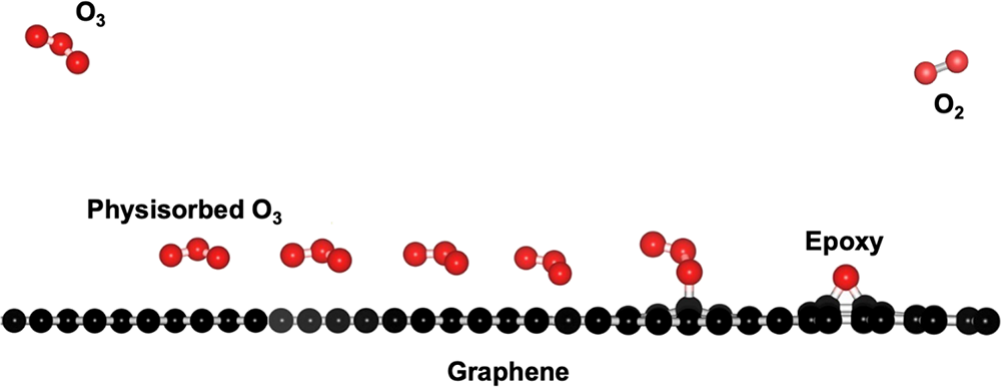

The exposure of graphene to O_3_ results in functionalization of its lattice with epoxy, even at room temperature. This reaction is of fundamental interest for precise lattice patterning, however, is not well understood. Herein, using van der Waals density functional theory (vdW-DFT) incorporating spin-polarized calculations, we find that O_3_ strongly physisorbs on graphene with a binding energy of −0.46 eV. It configures in a tilted position with the two terminal O atoms centered above the neighboring graphene honeycombs. A dissociative chemisorption follows by surpassing an energy barrier of 0.75 eV and grafting an epoxy group on graphene reducing the energy of the system by 0.14 eV from the physisorbed state. Subsequent O_3_ chemisorption is preferred on the same honeycomb, yielding two epoxy groups separated by a single C–C bridge. We show that capturing the onset of spin in oxygen during chemisorption is crucial. We verify this finding with experiments where an exponential increase in the density of epoxy groups as a function of reaction temperature yields an energy barrier of 0.66 eV, in agreement with the DFT prediction. These insights will help efforts to obtain precise patterning of the graphene lattice.

## Introduction

Controlled incorporation of defects in graphene by surface functionalization is attractive for modulating its electronic,^1,2^ magnetic,^3^ sorption,^4,5^ diffusion,^6−9^ and catalytic properties.^10,11^ Among various functional groups, the epoxy group, which is essentially an O atom bonded to two neighboring C atoms without breaking the C–C bond, is of fundamental interest. Epoxy groups alter the local density of states of graphene, which can be used for stepwise tuning of band gap of graphene.^12^ When multiple epoxy groups are grafted, they tend to form energy-minimizing clusters^13−15^ known to be responsible for the oxidative cleavage of graphitic lattice^16−18^ and the formation of vacancy defects.^19−21^ The formation of such defects, if controlled precisely, can be extremely attractive for application in molecular separation,^9,22−24^ enhanced water evaporation,^25^ sensing,^26,27^ sequencing,^28^ and power generation.^29^ In fact, exposure of graphene to O_3_ has become a promising way to prepare porous graphene membrane.^30−32^ However, the mechanism of the formation of the epoxy group from O_3_ is not well understood. An accurate description of underlying energetics would allow one to model and predict the evolution of epoxy groups and related clusters and will help efforts to precisely pattern graphene.

Theoretical studies on the interaction of O_3_ with graphene and carbon nanotubes are scarce, and report O_3_ physisorption energy in the range of −0.2 to −0.3 eV.^33−35^ The study of Lee and co-workers on the interaction of O_3_ with graphene is the most comprehensive report so far.^36^ They used the local spin density approximation (LSDA) to understand the reaction of O_3_ with graphene. They found that the O_3_ orientation on top of the graphene strongly influences its physisorption. However, the reported physisorption and chemisorption energies were small, −0.25 and −0.33 eV, respectively. Such moderate binding energies do not explain the strong affinity of O_3_ toward graphene or carbon nanotubes, which is observed by experiments at room temperature.^37,38^

Herein, using vdW-DFT calculations,^39^ we show that O_3_ physisorption on graphene is quite strong corresponding to a high binding energy of −0.46 eV. The interaction is uniquely maximized when O_3_ approaches graphene in a tilted (11° with respect to the horizontal plane), face-down orientation and when the terminal O atoms of O_3_ are positioned directly above neighboring graphene honeycomb. Ozone then goes through dissociative chemisorption to functionalize an epoxy group on graphene surpassing an energy barrier of 0.75 eV. The resulting chemisorption binding energy is high, 0.60 eV. We show that the second chemisorption event preferentially takes place on the same honeycomb, and the resulting pair of epoxy groups is separated by a single C–C bridge. Finally, we carry out experiments probing reaction of O_3_ with graphene. Monitoring the coverage of the epoxy group on graphene as a function of temperature, we calculate an energy barrier of 0.66 eV, in agreement with the DFT calculations.

## Experiments

### Theoretical calculations

To investigate O_3_ adsorption on graphene, vdW-DFT calculations were performed using the Quantum ESPRESSO package.^40,41^ A supercell made of 7 × 7 periodic unit cells of graphene was used to ensure the decoupling of in-plane molecule–molecule interactions. The Brillouin zone was sampled with uniform 4 × 4 × 1 unshifted k-point grids. A vacuum region of 20 Å was used in the *z*-direction to avoid interactions among the periodic replicas. An energy cutoff of 90 Ry was used for the plane wave expansion of the wave functions. A kinetic energy cutoff of 720 Ry on the charge was used together with ultrasoft pseudopotentials.^42,43^ In order to include the contributions of dispersion interactions, vdW-DF2^39^ approximation was used. For comparison, some adsorption energies were also calculated with the more recent, semiempirical vdW-DF3 approximation.^44^ Convergence thresholds of 2 × 10^–6^ Ry and 10^–4^ Ry/Bohr for the total energy and forces, respectively, are used.

For studying the dissociative chemisorption of O_3_ into epoxy and oxygen, spin-polarized calculations were performed. The reaction mechanisms and transition-state energy barriers were studied with climbing image nudged elastic band (NEB) calculations. The NEB simulations were carried out using a 6 × 6 supercell and a uniform 4 × 4 × 1 unshifted k-point grid to sample the Brillouin zone. The spin of the system is changed from the spin-neutral (O_3_) to a spin-polarized (epoxy + O_2_) at the transition state.

### Graphene Growth

To synthesize single-layer graphene, low-pressure chemical vapor deposition was used on annealed Cu foil with a thickness of 50 μm and a purity of 99.9% (Strem). The Cu foil was first annealed in an atmosphere of CO_2_ and H_2_ at 1000 °C for 30 min. The next step involved introducing CH_4_ and H_2_ gases with a 3:1 volume ratio into the reactor to initiate graphene growth. The reactor was maintained at 460 mTorr for 30 min during the growth process. Once the synthesis was complete, the reactor was rapidly cooled down to room temperature.

### Ozone Functionalization

Freshly synthesized graphene samples were functionalized by placing them in a quartz tube connected to an O_3_ generator yielding O_3_ concentration of 150 g/Nm^3^ in O_2_. The samples were heated to temperatures ranging from 290 to 329 K for functionalization. The reactor pressure was kept at 1 bar during each oxidation process. Following the functionalization, O_3_ was purged by flowing Ar. Finally, the samples were transferred to the ultrahigh vacuum (UHV) chamber of X-ray photoemission spectroscopy (XPS) for the characterization of the functional groups.

### XPS

High-resolution XPS measurements were conducted using a Kratos Analytical Axis Supra instrument equipped with the monochromated Kα X-ray line of an aluminum anode. The pass energy and step size used in this study were 20 and 0.1 eV, respectively. To prevent charge buildup, the sample was ground during measurements. The binding energy data were used without any corrections, and data processing was performed using CasaXPS software. Background subtraction was performed by using the Shirley method.

## Results and Discussion

**Figure 1:**
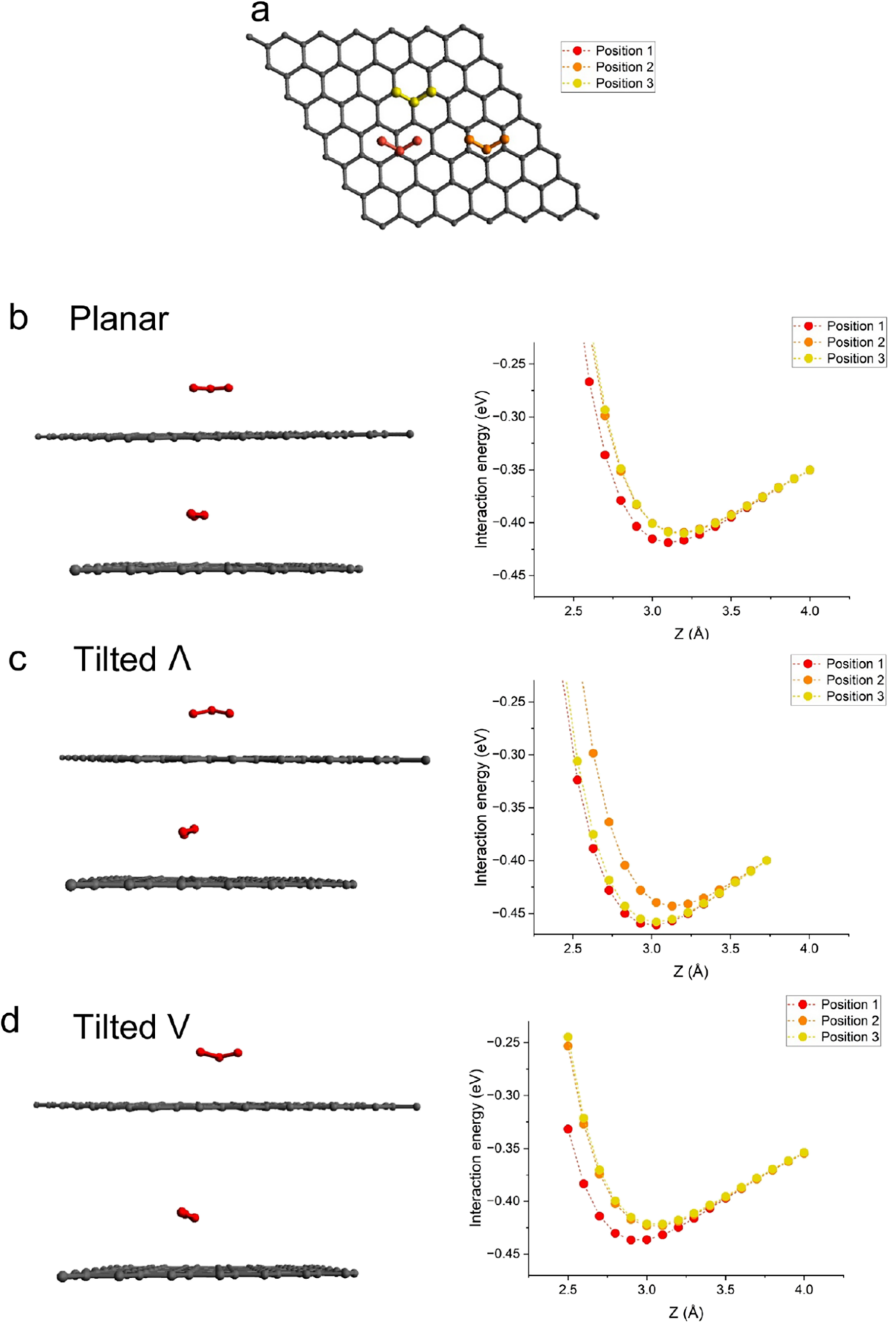
(a) Top view of the three O_3_ positions above the graphene lattice. Positions 1, 2, and 3 are highlighted by red, orange, and yellow, respectively. (b,d) Adsorption behavior of the graphene lattice of O_3_ as a function of positions (1, 2, and 3) and configurations (planar, tilted Λ, and tilted V) on top of the graphene lattice.

To study the physisorption behavior of the graphene lattice, the O_3_/graphene system was allowed to relax. O_3_ was relaxed in all directions to minimize the potential energy. C atoms in graphene were also relaxed to adjust their positions depending on the interactions with O_3_. To extract the lowest energy O_3_/graphene configuration, we explored several O_3_ configurations and positions on top of the lattice (Figure 1a). Briefly, the graphene lattice was placed in the *xy* plane at *z* = 0, and the pyrolytic element O_3_ was placed above graphene. Three orientations of the O_3_ were considered. These are a planar (horizontal) (Figure 1b) and two tilted vertical [face down or Λ (Figure 1c) and face up or V (Figure 1d)] configurations. In the vertical configurations, O_3_ was allowed to tilt to find the minimum energy configuration at a given *z*. We found that a tilt angle of 79° with respect to vertical (11° with respect to horizontal) was the most optimal configuration that minimized the energy as it allowed improved interaction for all three O atoms. Therefore, we fixed the tilt angle to 79° for the two tilted configurations. Finally, the relative distance between the central O of O_3_ and C atoms in graphene honeycomb was varied along the *xy* plane to probe three most probable positions (referred as position 1, 2, and 3 indicated by red, orange, and yellow, respectively in Figure 1a). In positions 1 and 3, the middle O of the O_3_ is on top of a C atom but with the following difference. In position 1, the terminal O atoms are near the middle of neighboring honeycombs. In position 3, the terminal O atoms are near the top of C atoms. In position 2, the terminal O atoms are placed on top of the center of the C–C bond.

The interaction energy as a function of O_3_-graphene distance (*z*) for the total nine configurations [positions 1, 2, and 3 in each three orientation (planar, tilted Λ, and tilted V)] are reported in Figure 1b–d. The distance refers to the *z* position of the O atom that is nearest to graphene. In all cases, as O_3_ is brought closer to graphene from 4 Å, the energy of the system first decreases and then increases. This is attributed to the increasing attractive interactions at first followed by the dominance of the repulsive dispersion interactions. Irrespective of the three orientations, the minimum energy is always obtained for the position 1 [red curve in the interaction energy plot, Figure 1b–d]. This is because in this position, terminal O atoms of O_3_ are surrounded by six C atoms of the honeycomb, which maximizes the interaction. In other words, the number of nearest neighbors (C atoms) for the terminal O atoms are highest in position 1, which ensures maximum interaction. Therefore, position 1 is the most favorable.

Final configurations are ranked as following in the order of their preference at the most optimal position, i.e., position 1: tilted Λ (−0.46 eV) > tilted V (−0.44 eV) > horizontal (−0.42 eV). Based on this, we assign the physisorbed state to position 1 in a tilted Λ configuration with physisorption binding energy of −0.46 eV. In this position, the terminal O of O_3_ is 3 Å away from the graphene lattice while the middle O is 3.3 Å away from the lattice (Figure 3a). When calculations were repeated with vdW-DF3, the physisorption binding energy at the same site was found to be −0.56 eV (−0.58 eV) using the OPT1 (OPT2) variant of the functional. We also calculated the change of zero potential energy (ZPE) and rotational + vibrational entropies upon adsorption (see calculations details in Supplementary Note S2). The resulting free energy correction is +0.09 eV, bringing the free energy of adsorption to values of −0.37 eV (vdW-DF2), −0.47 eV (vdW-DF3-OPT1), and −0.49 eV (vdW-DF3-OPT2).

The predicted physisorption energy in our study (−0.46 eV) is higher than reported by the literature^33−35^ and can explain the observed strong affinity of O_3_ toward graphene. We attribute our results to improved capture of long-range interactions, which allows maximization of interaction with all three O atoms. This perhaps also explains why the optimal configuration is close to planar with a slight (11°) tilt. In general, nonlocal density functionals, including the vdW-DF2 functional, are considered promising to model vdW interactions. They include nonlocal, long-range correlation interactions directly in the form of the potential of the density functional. To demonstrate the importance of vdW approximations, we also calculated the adsorption energy at the Perdew–Burke–Ernzerhof (PBE) level, which is less accurate in capturing the long-range interactions. PBE calculations indeed predicted a lower binding energy, −0.37 eV (see relaxed structure in Figure S1). While it also predicted the Λ configuration, O_3_ is not tilted (i.e., is in the vertical configuration) and the adsorption height is larger, 3.3 Å similar to what has been reported in the literature.^36^ We also studied the effect of the supercell size. A 3 × 3 supercell used in the literature is not large enough to completely remove the interactions between O_3_ molecules in neighboring images. Indeed, the binding energy increases and converges to −0.46 eV when the supercell size is increased from 5 × 5 to 6 × 6 and then to 7 × 7 periodic unit cells (Figure S2). Taken together, these findings underscore the importance of considering nonlocal density functionals, optimal configuration (tilted Λ in this case), and large enough supercell size for accurate modeling of physisorption interactions.

**Figure 2:**
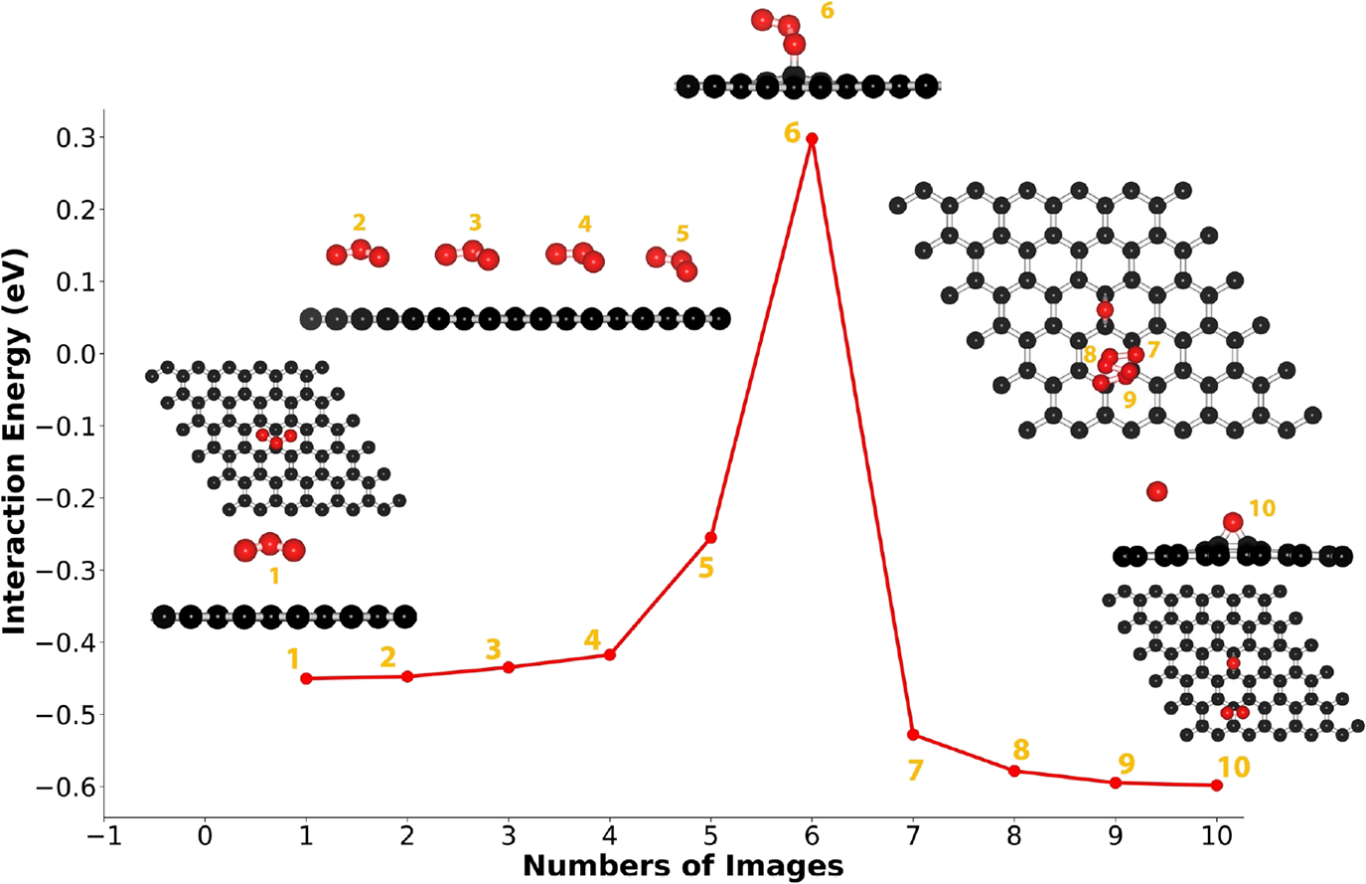
Interaction energy as a function of various configurations during chemisorption of O_3_ on graphene. The energy calculations are carried out by climbing image NEB calculations. The spin of the system is changed from the spin-neutral (O_3_) to a spin-polarized (epoxy + O_2_) at the transition state. All images are shown in detail in Figure S3.

Next, from the physisorbed O_3_, we carried out climbing image NEB calculations to understand the chemisorption of O_3_ on graphene (Figure 2). Detailed images for the stages of chemisorption are provided in Figure S3. NEB calculations reveal the following:

In the physisorbed tilted Λ configuration, terminal O atoms are closest to the graphene lattice with a distance of 3 Å. Chemisorption proceeds with the approach of a terminal O toward the C atom of the graphene lattice (images 2 and 3 in Figure 2, also see Figure S3). At this point, the net energy of the system starts to increase because of an increased repulsive interaction. The distance between the terminal O and C continues to decrease (images 4 and 5) until it reduces to 2.3 Å where a covalent O–C bond is formed (image 6). The net energy of the system is the highest at this point (0.3 eV). Therefore, we assign this as the transition state corresponding to an energy barrier of 0.75 eV from the physisorbed state.
After reaching the transition state, the bond length between the central O of O_3_ and the terminal O bonded to C increases until it dissociates leading to the formation of O_2_ and an epoxy group on graphene (image 7). When O_2_ is detached from the terminal O of O_3_, it prefers to be magnetic (see discussion on spin-polarization later). Therefore, to accurately capture the system energy, we performed spin-polarized calculations after O_2_ detachment. The energy of the system reduces significantly at this point. Subsequently, O_2_ drifts aways from the epoxy group (images 8 and 9) and physisorbs on top of the graphene lattice in a horizontal configuration (image 10). This reduces the energy of the system, compared to the physisorbed state, by 0.14 eV. Therefore, we assign a chemisorption binding energy of −0.6 eV. With vdW-DF3, the chemisorption binding energy was found to be −0.69 eV (−0.66 eV) using the OPT1 (OPT2) variant of the functional. Moreover, the free energy correction is +0.12 eV (Supplementary Note S2), bringing the free energy of adsorption to values of −0.48 eV (vdW-DF2), −0.57 eV (vdW-DF3-OPT1), −0.54 eV (vdW-DF3-OPT2).

**Figure 3:**
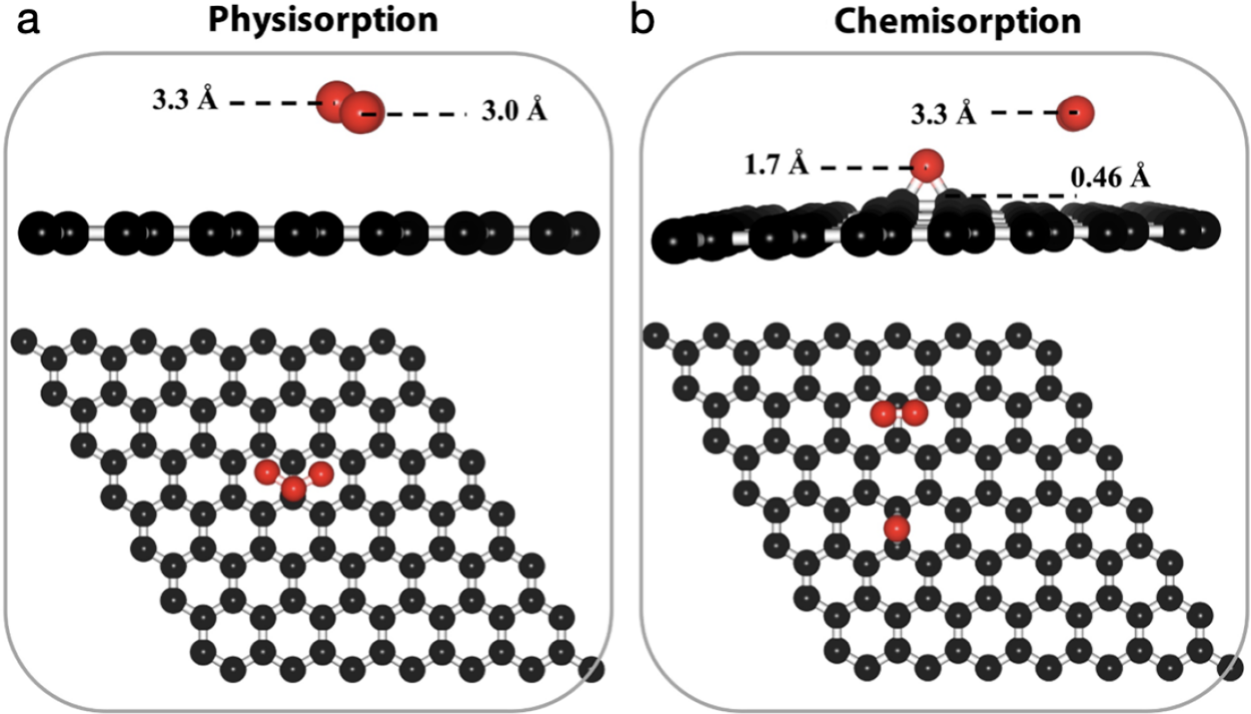
Physisorption (a) and chemisorption state (b) of O_3_ on graphene. The physisorption and chemisorption energies are −0.46 and −0.60 eV, respectively.

The physisorbed and chemisorbed states are compared in Figure 3. In the physisorbed state, the closest O of O_3_ is 3 Å away from the lattice (Figure 3a). In the chemisorbed state, O of the epoxy group is positioned ∼1.25 Å above the C–C bond in the epoxy group, maintained by the C–O bond which is 1.46 Å in length. The C atoms bonded epoxy are shifted out-of-plane by 0.46 Å which makes the O of the epoxy group close to 1.7 Å above the graphene plane. The dissociated O_2_ is also visible, which moves away from the epoxy group and is physisorbed on the lattice. The difference in the chemisorption energy also has a small contribution from the optimal configuration of dissociated O_2_ on graphene. The most optical configuration is when O_2_ is horizontally aligned on top of graphene at 3.3 Å away from the lattice (Figure 3b). This is in close agreement with the adsorption height of the standalone O_2_ molecule on graphene.^45−47^ This is also confirmed by a separate calculation for the adsorption of O_2_ on graphene with vdW-DF2 approximations (Figure S4). A projected density of states (PDOS) analysis is provided in Figures S7 and S8 of the Supporting Information to confirm the physisorbed/chemisorbed natures of the initial/final states, respectively.

As discussed above, during the transition state for chemisorption, the system undergoes a change from a spin-neutral system (O_3_) to a spin-polarized system (epoxy + O_2_). To reveal the importance of the spin-polarized calculation at the transition state, we also carried out the NEB calculations using a spin-neutral system (Figures S5 and S6). In this case, chemisorption is not favored compared to physisorption with an unfavorable binding energy. This underlines the importance of capturing the onset of spin in the O orbitals during the transition state for the chemisorption of the complexes of O_3_.

**Figure 4:**
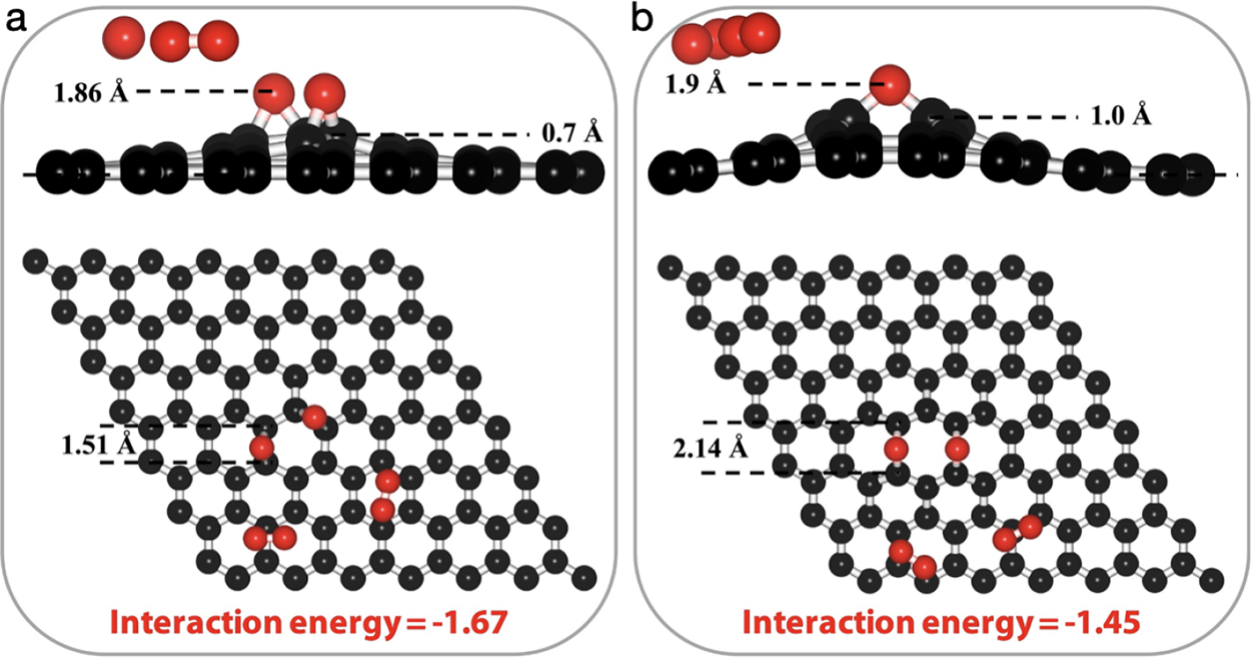
Comparison of the chemisorption of two O_3_ molecules in two potential configurations: (a) meta configuration and (b) para configuration.

It is reported that multiple epoxy groups on graphene lattice tend to cluster to minimize the energy of the system.^13,15,18,31^ As it has been shown that functional groups can pattern graphene,^48^ it is likely that epoxy group would promote the formation of another epoxy at a specific neighboring site. This can be attributed to the generation of electron deficiency on the neighboring C atom due to the more electronegative character of O. To understand the resulting configuration of the epoxy groups, we explored the energetics when an additional O_3_ reacts with graphene after a chemisorption event has already taken place. We considered two configurations with a pair of chemisorbed epoxy groups and a pair of physisorbed oxygen molecules from two consecutive chemisorption events (Figure 4).

In the first configuration, the epoxy groups are separated by a single bridge site and are located in the same honeycomb. We refer to this as the meta configuration (Figure 4a). In the second configuration, the epoxy groups are also located in the same honeycomb, but they are positioned opposite to each other. We refer to this as para configuration (Figure 4b). We find that the meta configuration is more favorable, as the system energy in this configuration (−1.67 eV) is lower by 0.22 eV than that in the para configuration (−1.45 eV). This finding is consistent with the literature report on cluster formation by the diffusion of epoxies.^18^ A likely reason for this is that the formation of the epoxy group leads to out-of-plane buckling of the lattice resulting in lattice strain. A configuration that reduces lattice strain should be preferred. Indeed, the out-of-plane buckling of the lattice in the meta configuration (0.7 Å) is lower than that in the para configuration (1.0 Å).

The net lower energy of the chemisorbed state compared to the physisorbed state indicates that O_3_ will prefer to chemisorb whenever it can overcome the energy barrier of 0.75 eV. The energy difference (0.14 eV) between the physisorbed and the chemisorbed states yields a large equilibrium constant (approximated as 227 at 25 °C, Supplementary Note S1) between the two states such that most of the sites on graphene would be in the chemisorbed state at the equilibrium.

The chemisorption time scale, τ_*c*_, can be predicted by the reciprocal of the chemisorption rate constant, *k*_*p*→*c*_, which in turn, can be approximated by the Eyring equation (eq 1):
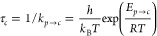
1where *E*_*p*→*c*_ refers to the energy barrier for chemisorption, *k*_B_ is the Boltzmann constant, *h* is Planck’s constant, and *R* is the universal gas constant. Based on this, τ_*c*_ at 25 °C is approximated as 0.7 s, i.e., one reaction nearly every second at room temperature. This explains the observed high reactivity of O_3_ with graphene where a significant coverage of epoxy groups on graphene have been reported upon short exposure of O_3_ at room temperature.^15^ This is also confirmed by experiments in this study (see below).

**Figure 5:**
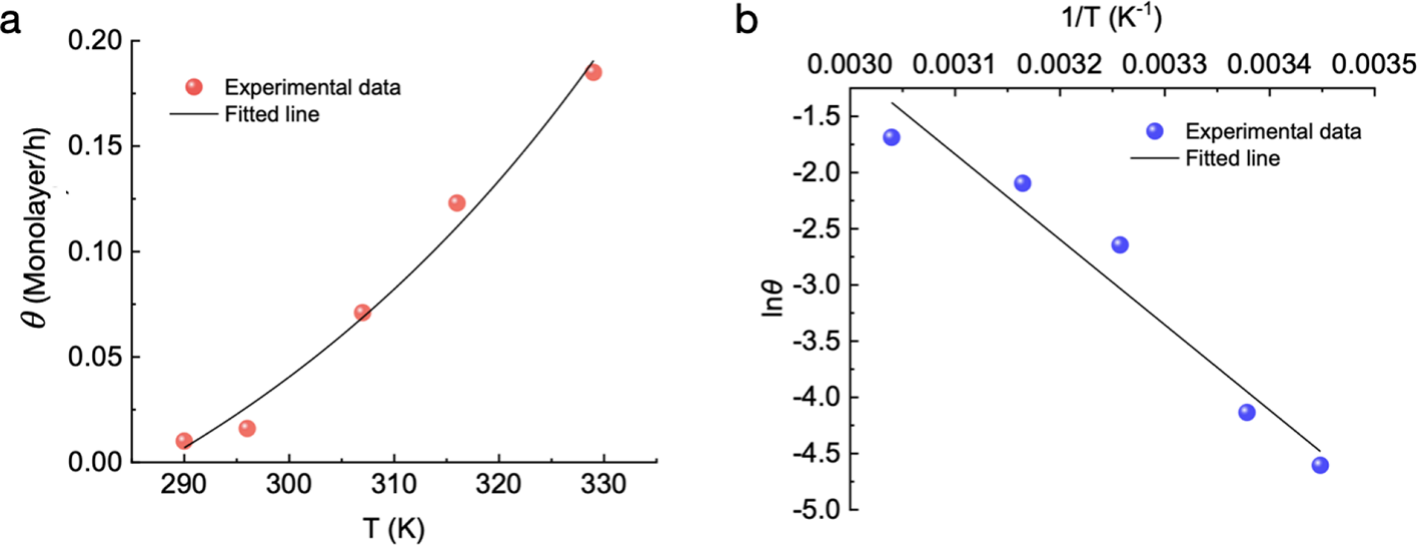
(a) Exponential fitting between the coverage rate (θ) of the epoxy groups on graphene and the reaction temperature (*T*). (b) Linear regression between ln θ and 1/*T*.

To obtain the chemisorption energy barrier by experiments, we prepared O_3_-functionalized graphene samples at various temperatures (17, 23, 34, 43, and 56 °C). The epoxy group coverage was analyzed by high-resolution XPS (Supplementary Note S3, Figures S9 and S10). For lower reaction temperatures (17 and 23 °C), we used a longer (12 h) reaction time to obtain a clear C–O peak in XPS for quantitative analysis. For samples prepared at or above 34 °C, 1 h of reaction was enough to obtain a clear C–O peak. The resulting coverage of epoxy group, indicated by the C 1s peak at 286.3 eV, was divided by the reaction time to obtain coverage rate, θ. We observed that θ increased exponentially in the probed temperature range from 0.01 monolayer/h at 17 °C to 0.016, 0.071, 0.123, and 0.185 monolayer/h at 23, 34, 43, and 56 °C, respectively (Figure 5a).

θ is driven by chemisorption whenever the chemisorption energy barrier, *E*_*p*→*c*_, is successfully overcome. At the initial stage of reaction (θ ≪ 1) where most site on graphene are available for reaction, θ is expected to be proportional to the chemisorption rate constant, *k*_*p*→*c*_, as per eq 2.
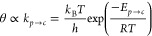
2

To extract *E*_*p*→*c*_ from the temperature-dependent data on θ, we plotted ln θ versus 1/*T* (Figure 5b). The obtained linear regression indicated an *E*_*p*→*c*_ of 0.66 eV, which agrees well with the vdW-DFT predicted energy barrier of 0.75 eV.

## Conclusions

Overall, we investigate the reaction of O_3_ with graphene using vdW-DFT. We find that O_3_ strongly physisorbs with a binding energy of −0.46 eV, maximized by the interaction of all three O atoms in the titled configuration with the nearest O atom 3 Å away from graphene. Chemisorption follows this forming epoxy group on graphene. The small energy barrier of chemisorption, 0.75 eV, makes O_3_ highly attractive to functionalize graphene even at room temperature, which is also demonstrated by experiments where a coverage rate of 0.071 monolayer/h is realized at 23 °C. From a computational point of view, we demonstrate the importance of nonlocal density functionals, which captures long-range interactions and improves the understanding of O_3_ sorption energetics and related configurations. We also demonstrate the importance of the spin-polarized system at the onset of the transition state where O_2_ dissociates from O_3_ to correctly capture the chemisorption energy. These calculations highlight that O_3_ is extremely attractive to functionalize graphene even at room temperature and can evolve as a popular tool to pattern the lattice of graphene for several applications relying on functionalized graphene.

## Supporting Information Available

Supporting Information references. The Supporting Information is available free of charge at https://pubs.acs.org/doi/10.1021/acs.jpcc.3c03994.

The O_3_ physisorption on a graphene plane at PBE level (Figure S1); O_3_ physisorption on graphene with different supercell size (Figure S2); all O_3_ configurations on graphene related to transition from the physisorption state to the chemisorption state (Figure S3); the physisorption state of O_2_ molecule on graphene (Figure S4); interaction energy as a function of various configurations during chemisorption of O_3_ on graphene when the spin of the system is maintained as spin-neutral (Figure S5); all configurations for O_3_ chemisorption when the spin of the system is maintained as spin-neutral (Figure S6); PDOS of the initial state of Figure 2 onto O_3_ orbitals (Figure S7); PDOS of the final state of Figure 2 (main text) onto O_2_ and O orbitals (Figure S8); high-resolution C 1s XPS spectra of the O_3_-functionalized graphene prepared at different temperatures (Figure S9); high-resolution C 1s XPS spectra of the H_2_-annealed Cu foil and O_3_-functionalized Cu foil prepared at 25 °C for 1 h (Figure S10); equilibrium constant between the adsorption and chemisorption states (Supplementary Note S1); free energy correction (Supplementary Note S2); determination of the coverage of epoxy group on graphene by XPS (Supplementary Note S3); the coverage of C–O and C=O groups on the treated Cu foils (Table S1); the coverage of C–O and C=O groups on the O_3_-functionalized graphene samples (Table S2) (PDF)
