# Supplementary material for: Mechanistic Insights on Functionalization of Graphene with Ozone
Source: J Phys Chem C Nanomater Interfaces. 2023 Nov 7;127(45):22015–22. doi: 10.1021/acs.jpcc.3c03994 (PMC10658624; doi:10.1021/acs.jpcc.3c03994)
Supplement: Supplementary file 1 — jp3c03994_si_001.pdf [file jp3c03994_si_001.pdf]

Supporting Information

Mechanistic Insights on Functionalization of Graphene with Ozone

Mohammad Tohidi Vahdat,[1,2†] Shaoxian Li,[1†] Shiqi Huang,[1] Luc Bondaz,[1] Nicéphore Bonnet,[2] Kuang-Jung Hsu,[1] Nicola Marzari,[2] Kumar Varoon Agrawal[1*]*

[1]Laboratory of Advanced Separations (LAS), École Polytechnique Fédérale de Lausanne (EPFL), CH-1950, Sion, Switzerland

[2]Theory and Simulation of Materials (THEOS) and National Centre for Computational Design and Discovery of Novel Materials (MARVEL), EPFL, CH-1015, Lausanne, Switzerland

[*]Correspondence: nicola.marzari@epfl.ch; kumar.agrawal@epfl.ch

[†]These authors contributed equally.

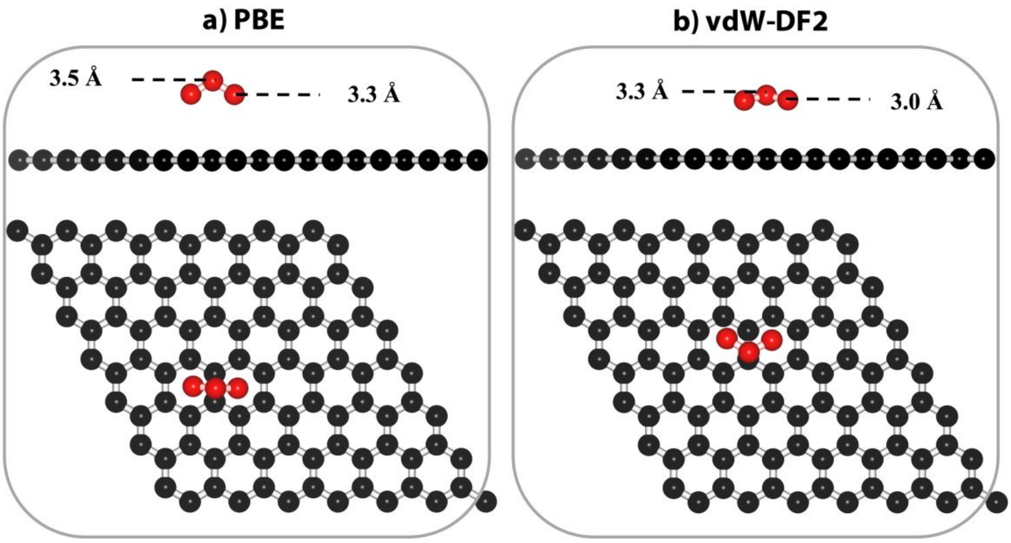

Figure S1. Optimal configurations for the adsorption of O_3 on graphene calculated by (a) at PBE level and (b) with the vdW-DFT approximation. The height of O atoms of O_3 above the graphene plane is mentioned in the side view.

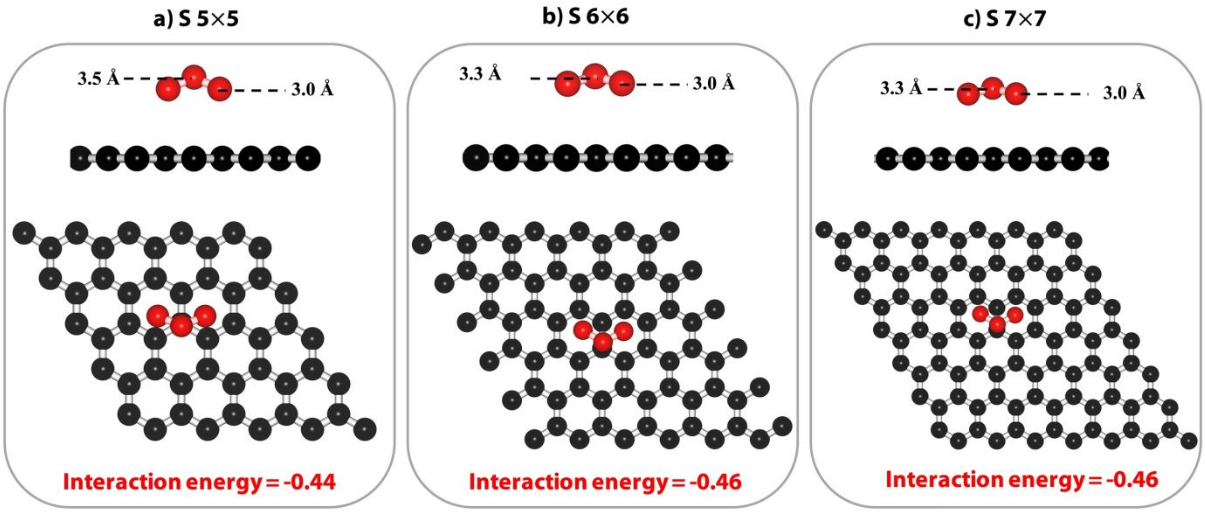

Figure S2. O_3 adsorption configuration and the corresponding adsorption height calculated using supercell made of (a) 5×5, (b) 6×6, and (c) 7×7 periodic unit cells of graphene with the vdW-DFT approximation.

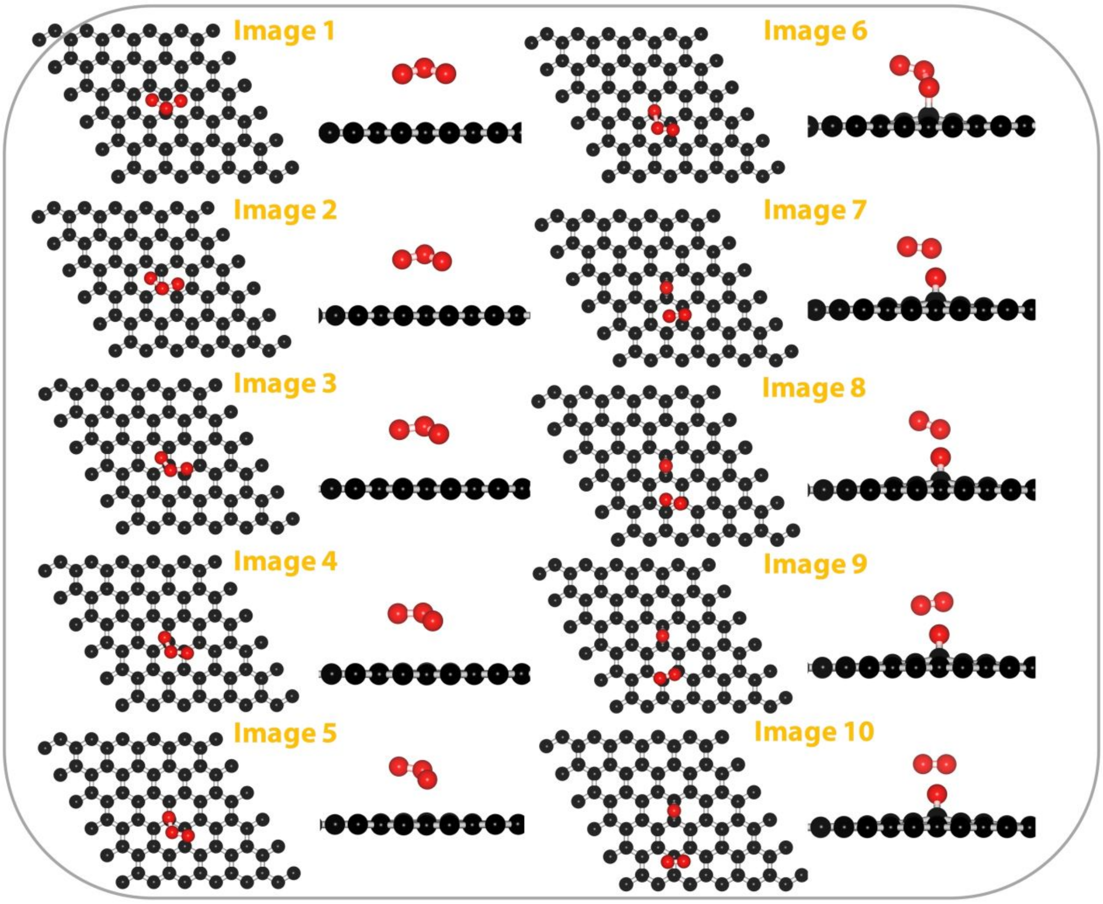

Figure S3. All the configurations encountered in O_3 chemisorption on graphene. The spin of the system is changed from the spin-neutral (O_3) to a spin-polarized (epoxy + O_2) at the transition state.

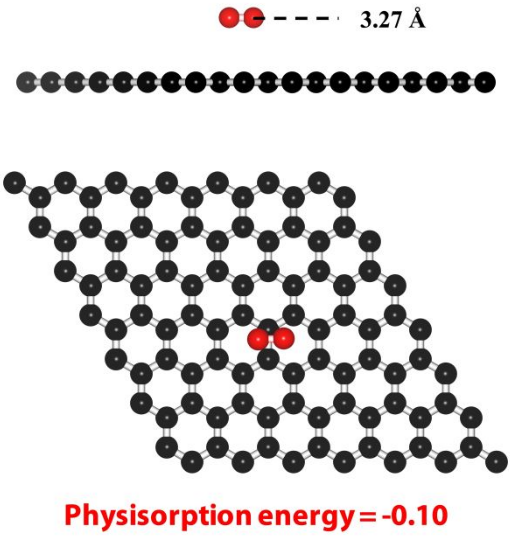

Figure S4. The final configuration of oxygen molecule at the adsorption state, which is calculated using vdW-DFT approximation. The adsorption height for the oxygen molecule is 3.27 Å. The physisorption energy for an oxygen molecule on graphene is -0.10 eV.

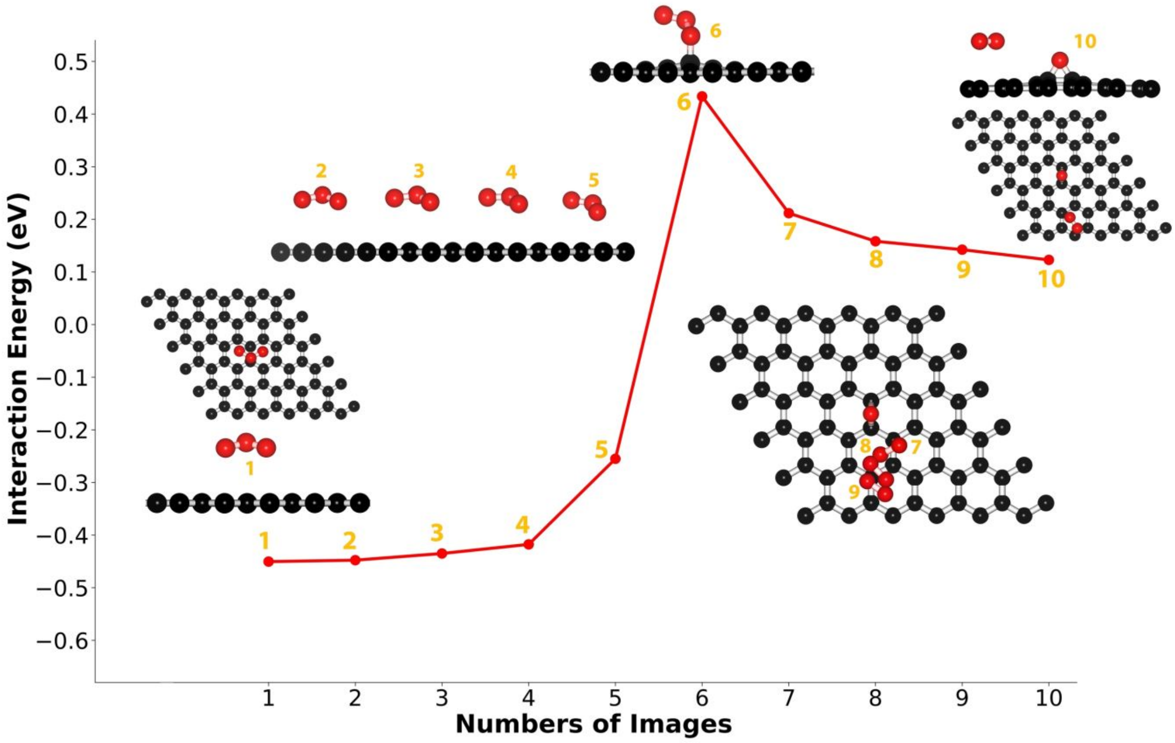

Figure S5. Interaction energy as a function of various configurations during chemisorption of O_3 on graphene when the spin of the system is maintained as spin-neutral. Further details of the configurations are shown in Figure S6.

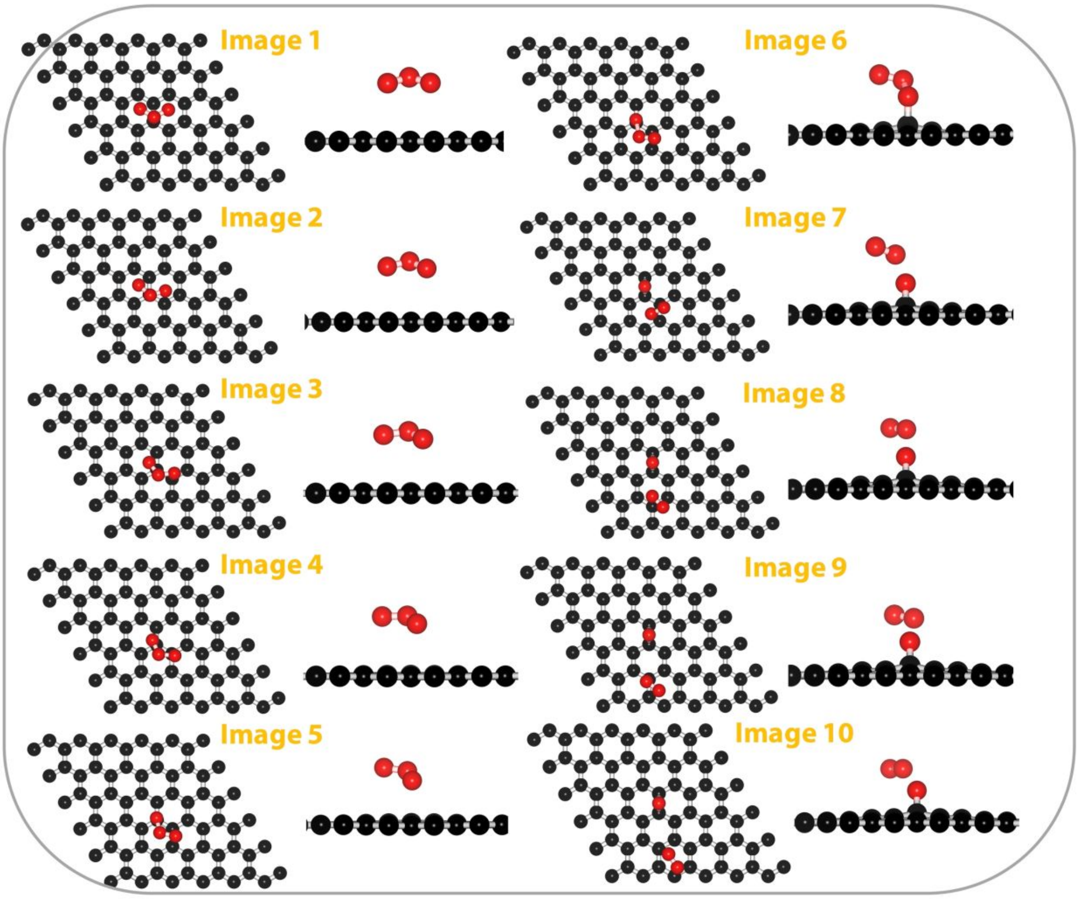

Figure S6. All the configurations encountered in O_3 chemisorption on graphene when the spin of the system is maintained as spin-neutral.

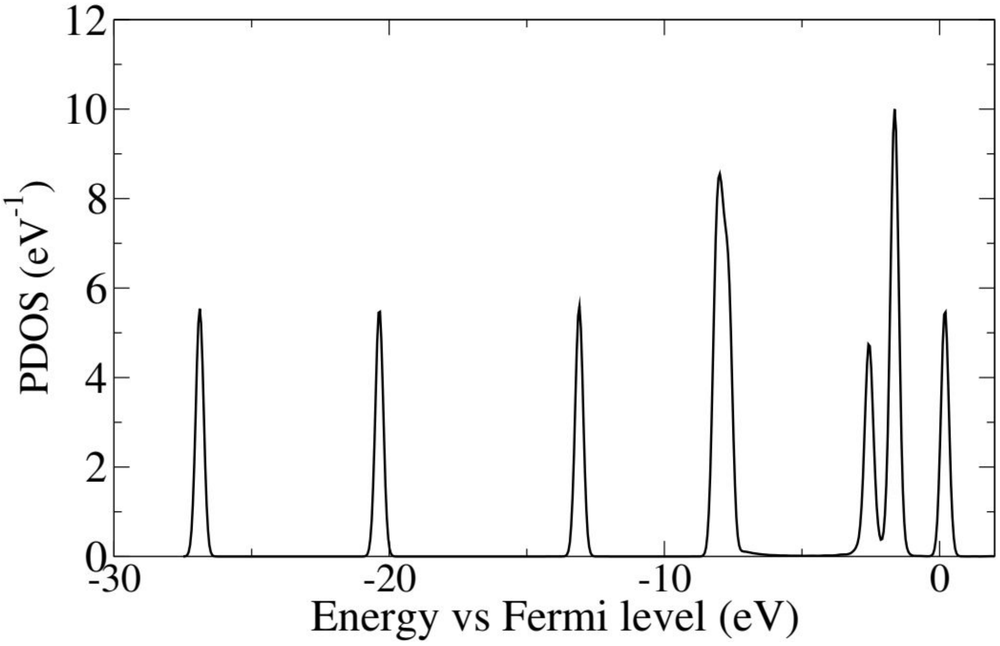

Figure S7. PDOS of the initial state of Figure 2 onto O_3 orbitals. The O_3 molecular peaks show no sign of hybridization, illustrating the physisorbed nature of O_3 on graphene.

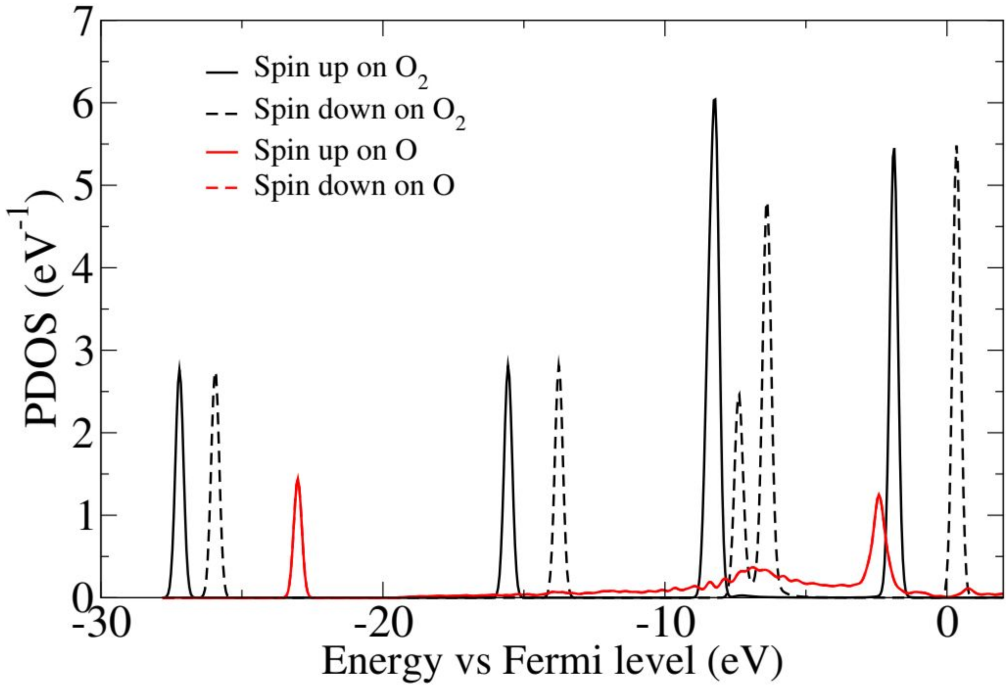

Figure S8. PDOS of the final state of Figure 2 onto O_2 and O orbitals. The O_2 molecular peaks show no sign of hybridization, illustrating the physisorbed nature of O_2 on graphene. By contrast, the strong hybridization of the single oxygen *p*-orbitals (resulting into the continuous PDOS spread between -15 and 0 eV vs the Fermi level) confirms the chemisorbed nature of the single O atom.

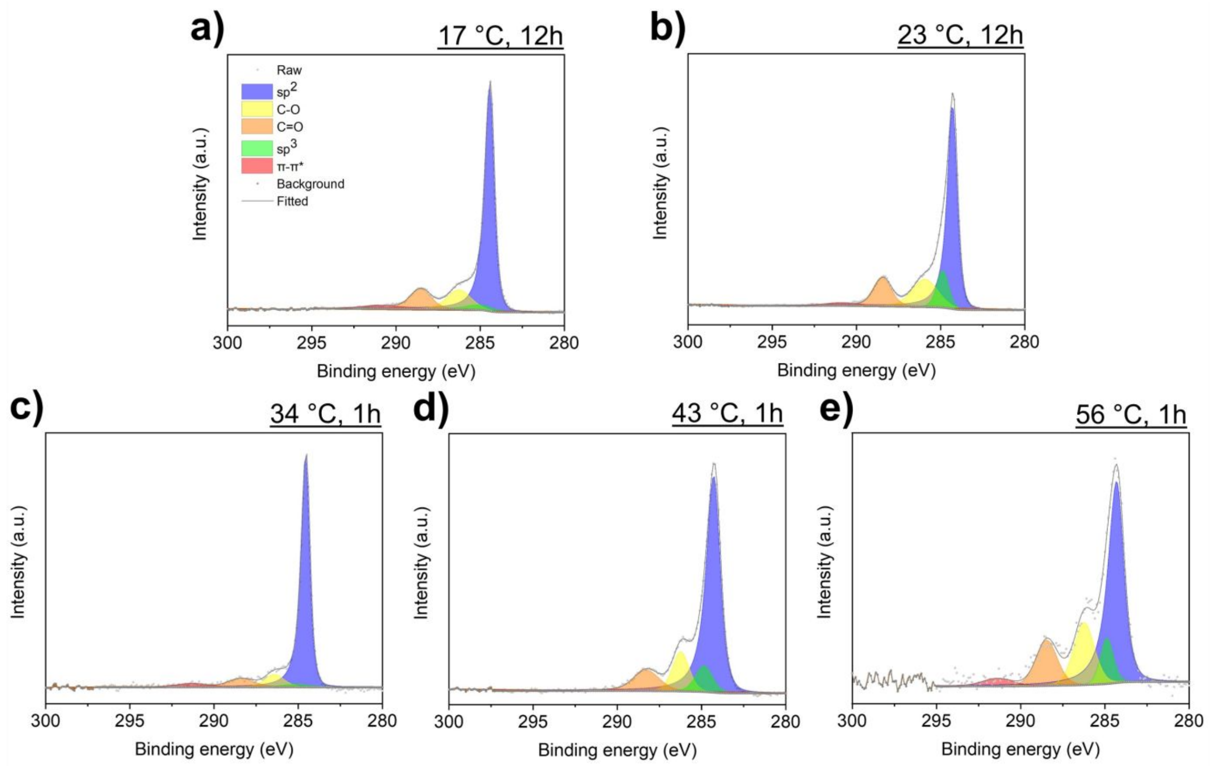

Figure S9. High-resolution C1s XPS spectra of the O_3-functionalized graphene prepared at 17 ºC for 12 h (a), 23 ºC for 12 h (b), 34 ºC for 1 h (c), 43 ºC for 1 h (d), and 56 ºC for 1 h (e).

Supplementary Note S1: Equilibrium constant between the adsorption and chemisorption states

Equilibrium constant for physisorbed and chemisorbed state can be expressed as following:

$$O_{3_{physisorbed}} \Leftrightarrow O_{3_{chemisorbed}}$$

where chemisorbed O_3 refers to the formation of an epoxy group and a physisorbed O_2.

The energy barrier for chemisorption from the physisorbed state, $E_{p\to c}$, is 0.75 eV (Figure 2 in main text). The energy barrier for the reverse path, $E_{c\to p}$, i.e., generation of the physisorbed state from the chemisorbed state is 0.89 eV (Figure 2, corresponding to the energy difference between transition state (image 6) and image 10). The equilibrium constant for the reaction (K_{eq} is given by ratio of forward ($k_{p\to c}$) to the backward ($k_{c\to p}$) rate constants as following (equation S1):

$$K_{eq} = \frac{k_{p\to c}}{k_{c\to p}} \qquad \text{(S1)}$$

$k_{p\to c}$and $k_{c\to p}$ can be approximated based on Eyring equation:

$$k_{p\to c} = \frac{k_B T}{h} exp\left(\frac{-E_{p\to c}}{RT}\right) \qquad \text{(S2)}$$

$$k_{c\to p} = \frac{k_B T}{h} exp\left(\frac{-E_{c\to p}}{RT}\right) \qquad \text{(S3)}$$

where k_B is the Boltzmann constant, h is the Planck's constant, T is the temperature, and R is the universal gas constant.

Combining equations (S1), (S2), and (S3), one obtains the following approximation:

$$K_{eq} = exp\left(\frac{-E_{p\to c} - E_{c\to p}}{RT}\right) \qquad \text{(S4)}$$

Substituting for the energy barriers, one obtains K_{eq} as 227 at 25 ºC.

Supplementary Note S2: Free energy correction

The rotational entropy of O_3 in the gas phase is explained by McQuarrie.[1]

$$S_{rot} = k_B\left[ln\left(\frac{\pi^{1/2}}{\sigma}\prod_{i=1}^{3}\left(\frac{8\pi^2 I_i k_B T}{h^2}\right)^{1/2}\right)+\frac{3}{2}\right] \quad (S5)$$

where I_i is the i-th moment of inertia of the molecule, and $\sigma = 2$.

The vibrational frequencies of the O_3 molecule are determined in the gas phase and its adsorbed (physisorbed/chemisorbed) states from the eigenvalues of the dynamical matrix calculated by finite displacements of O_3 atoms around the energy minimum. In the gas phase, the resulting vibrational frequencies are 1173, 1059, 700 cm^{-1}. For the physisorbed state (O_3 on graphene), we find frequencies of 1095, 1027, 672 cm^{-1} (corresponding to the same internal vibrational modes), 56 cm^{-1} (translation normal to the surface), 141, 79, 32 cm^{-1} (rotations). For the chemisorbed state (physisorbed O_2 + chemisorbed O), we find frequencies of 1575 cm^{-1} (O_2 stretching), 684, 510, 396 cm^{-1} (O vibrational modes), 41 cm^{-1} (O_2 translation normal to the surface), 91, 86 cm^{-1} (O_2 rotations). In each case, the two remaining modes have very low frequencies (<17 and <3 cm^{-1}) and correspond to the translation of O_3 and O_2 molecules in the plane parallel to the surface.

The zero-point energy (ZPE) is obtained as the sum $\sum_i \frac{h\nu_i}{2}$ over the vibrational frequencies ν_i.

The entropy of a vibrational mode ν is given by McQuarrie.[1]

$$S_{vib} = k\left[\frac{h\nu}{kT\left(e^{\frac{h\nu}{kT}}-1\right)} - ln\left(1-e^{\frac{-h\nu}{kT}}\right)\right] \quad (S6)$$

This formula is used for the internal molecular vibrations, the O vibrational modes, and molecular translations normal to the surface. However, for the remaining rotor-like vibrational modes (rotations of O_3 and O_2 on graphene), the expression introduced by Grimme[2] is used:

$$S = w(\nu)S_{vib} + (1-w(\nu))S_r \quad (S7)$$

with $S_r = k\left[ln\left(\left(\frac{8\pi^3\mu' kT}{h^2}\right)^{1/2}\right)+\frac{1}{2}\right]$; $\mu' = \frac{\mu I}{\mu + I}$; $\mu = \frac{h}{8\pi^2\nu}$; $w(\nu) = \frac{1}{\left(1+\left(\nu_0/\nu\right)\right)^{\alpha}}$ (S8)

where ν is the vibrational frequency, I the moment of inertia of the molecule around the rotation axis, ν_0 = 100 cm^{-1}, and α = 4.

Using this methodology, we find ZPEs for the gas-phase, physisorbed, and chemisorbed O_3 of 0.182, 0.193, 0.210 eV, and rotational + vibrational entropies times room temperature of 0.263, 0.186, 0.169 eV, respectively. The resulting free energy corrections are +0.09 and +0.12 eV for the physisorption and chemisorption energies, respectively.

Supplementary Note S3: Determination of the coverage of the epoxy group on graphene by XPS

In order to identify the functional group that was attached to the surface of the graphene, we performed control experiments involving O_3 functionalization on Cu foil that had been annealed in an H_2 atmosphere (Figure S10). This functionalization process took place at room temperature (25 ºC) for a duration of 1 hour, using an O_3 flow. This data (Figure S10) reveals excessive buildup of C=O group on Cu which is likely contamination.

For experiments done on the Cu foil, the coverages of C-O and C=O groups attached to the sample's surface are presented in Table S1. The data from this table reveals that the coverage of C-O remained relatively unchanged (from 7.3% to 7.8%), which can be attributed to the low coverage of the sp^2 carbon structure. However, we observed an increase of approximately 18.1% in the coverage of the C=O group following the O_3 treatment. This finding suggests that the contamination produced during the O_3 treatment predominantly consists of C=O groups, rather than C-O groups. Therefore, it can be inferred that the C-O groups formed on the graphene samples during the O_3 treatment correspond to epoxy groups that are covering the graphene surface. Conversely, the C=O groups, which exhibited the highest coverage of 14.1% (lower than 18.1%, as indicated in Table S2 at 56 °C), can be attributed to contamination. Our hypothesis is that C=O contamination mainly arrives from O-rings used to seal the reactor which are difficult to avoid.

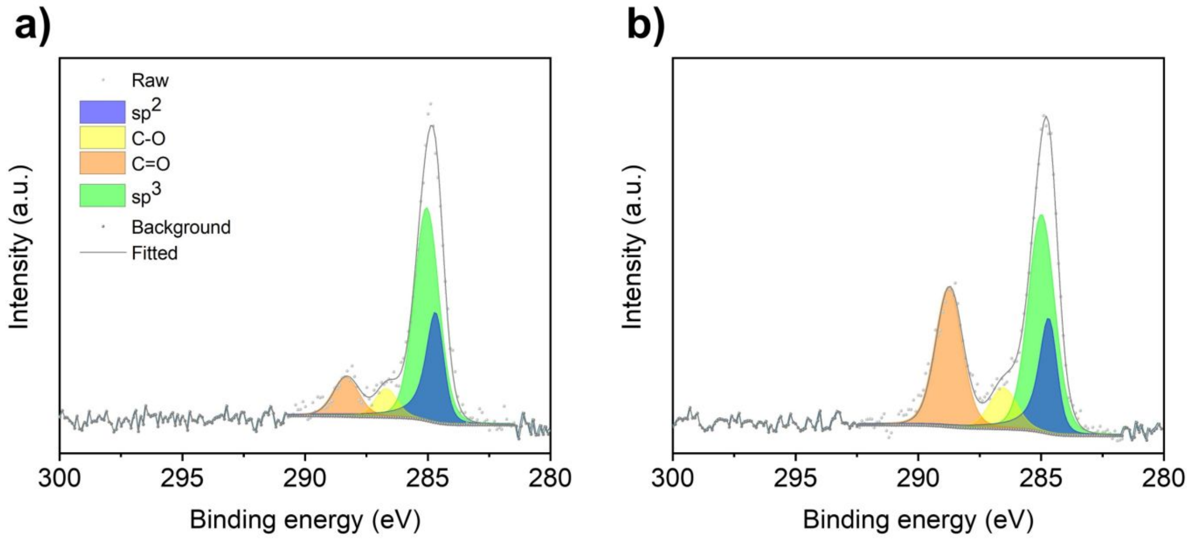

Figure S10. High-resolution C1s XPS spectra of the H_2-annealed Cu foil (a), and O_3-functionalized Cu foil prepared at 25 ºC for 1 h (b).

Table S1. The coverage of C-O and C=O groups on the treated Cu foils.

Treatment	C-O coverage (%)	C=O coverage (%)
H_2-annealed Cu foil	7.3	10.9
O_3-functionalized Cu foil at room temperature for 1 h	7.8	29

Table S2. The coverage of C-O and C=O groups on the O_3-functionalized graphene samples.

Temperature (°C)	C-O coverage in 1 h (%)	C=O coverage in 1 h (%)
17	1	0.9
23	1.6	1
34	7.1	6.9
43	12.3	9.9
56	18.5	14.1

References

(1) McQuarrie D.A. *Statistical Mechanics*; Harper and Row Publishers Inc., 1976.

(2) Grimme, S. Supramolecular Binding Thermodynamics by Dispersion-Corrected Density. *Chem. Eur. J.* **2012**, *18*, 9955–9964. https://doi.org/10.1002/chem.201200497.
